# Evaluation of loop-mediated isothermal amplification as a surveillance tool for malaria in reactive case detection moving towards elimination

**DOI:** 10.1186/s12936-018-2399-x

**Published:** 2018-07-09

**Authors:** Munyaradzi Tambo, Joyce R. Auala, Hugh J. Sturrock, Immo Kleinschmidt, Ronnie Bock, Jennifer L. Smith, Roland Gosling, Davis R. Mumbengegwi

**Affiliations:** 1 Multidisciplinary Research Center, University of Namibia, Windhoek, Namibia; 2 Department of Biological Sciences, University of Namibia, Windhoek, Namibia; 3 Malaria Elimination Initiative, Global Health Group, University of California San Francisco, San Francisco, CA USA; 4 MRC Tropical Epidemiology Group, Department of Infectious Disease Epidemiology, London School of Hygiene and Tropical Medicine, London, UK

## Abstract

**Background:**

As malaria transmission decreases, the proportion of infections that are asymptomatic at any given time increases. This poses a challenge for diagnosis as routinely used rapid diagnostic tests (RDTs) miss asymptomatic malaria cases with low parasite densities due to poor sensitivity. Yet, asymptomatic infections can contribute to onward transmission of malaria and therefore act as infectious reservoirs and perpetuate malaria transmission. This study compared the performance of RDTs to loop-mediated isothermal amplification (LAMP) in the diagnosis of malaria during reactive active case detection surveillance.

**Methods:**

All reported malaria cases in the Engela Health District of Namibia were traced back to their place of residence and persons living within the four closest neighbouring houses to the index case (neighbourhood) were tested for malaria infection with RDTs and dried blood spots (DBS) were collected. LAMP and nested PCR (nPCR) were carried out on all RDTs and DBS. The same procedure was followed in randomly selected control neighbourhoods.

**Results:**

Some 3151 individuals were tested by RDT, LAMP and nPCR. Sensitivity of RDTs and LAMP were 9.30 and 95.50%, respectively, and specificities were 99.27 and 99.92%, respectively, compared to nPCR. LAMP carried out on collected RDTs showed a sensitivity and specificity of 95.35 and 99.85% compared to nPCR carried out on DBS. There were 2 RDT samples that were negative by LAMP but the corresponding DBS samples were positive by PCR.

**Conclusion:**

The study showed that LAMP had the equivalent performance as nPCR for the identification of *Plasmodium falciparum* infection. Given its relative simplicity to implement over more complex and time-consuming methods, such as PCR, LAMP is particularly useful in elimination settings where high sensitivity and ease of operation are important.

## Background

As countries set goals to eliminate malaria, detecting a large fraction of infections becomes increasingly important in order to interrupt transmission [1]. Detecting symptomatic cases is feasible because patients generally have high-density infections and present to health facilities passively where they can be detected by rapid diagnostic test (RDT). However, detecting asymptomatic infections in the community is a challenge during passive and reactive case detection of malaria because asymptomatic individuals do not seek treatment. These infections are often low density and below the threshold of detection for microscopy and RDTs, yet can continue to infect mosquitoes and sustain transmission [2, 3]. This is a particular challenge for low transmission settings where evidence suggests that sub-patent infections can comprise 70–80% of all malaria infections [4].

Active case detection, whereby infections are actively sought out and treated within the community, becomes a recommended approach [1]. Namibia has recently begun reactive active case detection (RACD) which involves testing and treating individuals living in close proximity to reported cases because malaria cases can be geographically clustered around the reported cases [1, 5].

Asymptomatic infections are however usually associated with sub-patent infections, below the density detectable (< 50 parasites/µL) by current RDTs [2]. Detection of these infections relies on more sensitive methods, such as polymerase chain reaction (PCR), which have a detection limit of 1–2 parasites/µL when extracted from a dry blood spot (DBS) [4, 6, 7]. While PCR is often considered the gold standard method of detection, it requires substantial technical expertise and infrastructure, limiting its operational use. An alternative method recently developed is loop-mediated isothermal amplification (LAMP). LAMP has been shown to have excellent diagnostic performance and is simpler to perform. A number of studies have reported that LAMP could easily be scaled up as it requires minimal training and equipment and has a short turnaround time for results (45 min) [8]. LAMP has been reported to have a sensitivity highly consistent with nPCR and there are reports of LAMP being more sensitive than PCR [8–10]. Both LAMP and PCR have a high sensitivity as a result of the amplification of the detection signal, DNA.

The diagnostic accuracy of LAMP was compared to RDT using nPCR as a gold standard, in a low transmission setting of northern Namibia. In addition, blood samples from RDT were tested to determine if they can provide sufficiently high quality blood samples for molecular analysis to those collect as DBSs.

## Methods

This study was part of a wider epidemiological study in northern Namibia. Details of this study are presented elsewhere [11]. Briefly, all consenting individuals living within households of cases passively detected at any one of the 17 health facilities in Engela Health district, in Ohangwena region of Namibia had blood taken for a RDT and a DBS. The blood samples were collected from consenting individuals living within four neighbouring households of index cases, including the index case household. A total of 2642 RDT and DBS samples were collected. The RDT and DBS samples were stored in labelled, ziplock bags with desiccant at − 20 °C at a health facility in the field. These samples were transported to the laboratory on ice where they were stored (at − 20 °C) then processed. LAMP was run on all RDT and DBS samples. nPCR was run on all LAMP and RDT positive samples and 10% of the LAMP negative samples using DBS as the source of DNA.

### Chelex DNA extraction

DNA was extracted from the collected RDT and DBS samples using the chelex extraction method. The RDT cassette was opened with a surgical blade and a total of 4 pieces of similar size were cut from the nitrocellulose strip inside the RDT cassette with a surgical blade that was sterilized with ethanol and washed with water after each use. The DNA was concentrated between the control line and the blood loading point, the 4 pieces were cut from this section. There were 4 DBSs on each DBS sample; a small circular piece (≈ 5 mm) in diameter was cut from one of the 4 DBSs on the filter paper using a puncher that was sterilized with ethanol and washed with water between samples.

### LAMP

In this procedure, Pan-LAMP tubes (able to detect all 4 species of *Plasmodium* that infect humans) were used. The kits were used according to the manufacturer’s protocol (LMC 562, Eiken Chemical Co Ltd, Tokyo, Japan). LAMP was performed to determine the presence of *Plasmodium* parasites in the blood samples based on the presence or absence of *Plasmodium* DNA.

Cytochrome B nested-PCR targeting the genus *Plasmodium* (*Plasmodium falciparum, Plasmodium ovale, Plasmodium malariae, Plasmodium vivax*) was performed on every positive RDT and LAMP sample and on every 10th negative sample as a reference for quality assurance of DNA isolation of positive and negative results. The nested-PCR was run using the primers CB1 and CB2 for the primary round, and NCB1 and NCB2 were used for the nested round with the diluted product from the first round being used as a template as shown in Table 1. Cytochrome B nested-PCR is highly sensitive in detecting *P. falciparum* infections that are dominant in Namibia. The PCR conditions were as shown in Table 2. After the nested round of PCR, 5 µL of the PCR product were mixed with 2 µL of loading dye. These samples were then loaded onto a 2% polyacrylamide gel and run for 110 min at 90 volts. After running the gel, it was placed in a gel documenting system that was connected to a desktop computer to visualize the results.

**Table 1:** Primer sequences for LAMP amplification of the *Plasmodium* genus for detection of *Plasmodium falciparum, Plasmodium vivax, Plasmodium ovale* and *Plasmodium malariae*

Species	Primer	Primer sequence (5′ to 3′)
*P. falciparum*, *P. vivax, P. ovale, P. malariae)*	Forward Inner Primer (F1P) (F1c + F2 regions)	AGCTGGAATTACCGCGGCTGGGTTCCTAGAGAAACAATTGG
	Backward Inner Primer (B1P) (B1-B2c regions)	TGTTGCAGTTAAAACGTTCGTAGCCCAAACCAGTTTAAATGAAAC
	Forward Outer Primer (F3) (F3c + F3 region)	TGTAATTGGAATGATAGGAATTTA
	Backward Outer Primer (B3) (B3 + B3c region)	GAAAACCTTATTTTGAACAAAGC
	Loop Forward Primer (LPF)	GCACCAGACTTGCCCT
	Loop Backward Primer (LPB)	TTGAATATTAAAGAA

**Table 2:** Cycling conditions for amplification of the PCR primary and secondary round

PCR conditions	
Primary round PCR programme: 3 h	Nested round PCR programme: 3 h
1. Initial denaturation—94 °C × 5 m	1. Initial denaturation—94 °C × 5 m
2. 40 Cycles—94 °C × 30 s, 52.5 °C × 90 s, 68 °C × 90 s	2. 40 Cycles—94 °C × 30 s, 60 °C × 90 s, 72 °C × 90 s
3. Final elongation—68 °C × 10 m	3. Final elongation—72 °C × 10 m
4. Hold at 4 °C	4. Hold at 4 °C

### Data analysis

Medcalc^®^ statistical software was used to assess diagnostic performance of RDTs compared to LAMP. The diagnostic performance test calculates 4 parameters, which are sensitivity, specificity, positive predictive value (PPV), and negative predictive value (NPV).

## Results

Out of 2642 people screened 2642 had RDTs and 2640 DBS available for assessment. LAMP was carried out on 2642 RDTs and 2640 DBS samples. nPCR was carried out on 56 DBS samples corresponding to all positive samples by RDT and/or LAMP and 10% of negative DBS (Table 3).

**Table 3:** Diagnostic evaluation test of LAMP and RDT with nPCR as the gold standard

	Total number tested	Number positive (%)	Number negative (%)	Sensitivity 95% confidence interval	Specificity 95% confidence interval	Positive predictive value 95% confidence interval	Negative predictive value 95% confidence interval
RDTs	2642	23	2619	9.30% (2.59–22.14%)	99.24% (98.86–99.56%)	17.39% (6.96–37.22%)	98.51% (98.36–98.65%)
LAMP extracted from RDT	2642	45	2597	95.35% (84.19–99.43%)	99.85% (99.61–99.96%)	99.11% (79.34–96.47%)	99.92% (99.70–99.88%)
LAMP extracted from DBS	2640	47	2593	95.50% (84.85–99.46%)	99.92% (99.72–99.99%)	95.56% (84.31–98.85%)	99.92% (99.70–99.98%)
nPCR extracted from DBS	315	43	262	Reference	Reference	Reference	Reference

*RDTs* rapid diagnostic test, *LAMP* loop-mediated amplification, *DBS* dried blood spot, *nPCR* nested polymerase chain reaction

There were 23 RDT detected malaria infections from a total of 2642 individuals. The sensitivity, specificity, PPV and NPV of RDTs were 9.30, 99.27, 17.39, and 98.51%, respectively.

There were 45 detected malaria infections with LAMP using RDT as the DNA source. These were detected with sensitivity, specificity, PPV and NPV of 95.35, 99.85, 91.11, and 99.92%, respectively.

**Fig. 1:**
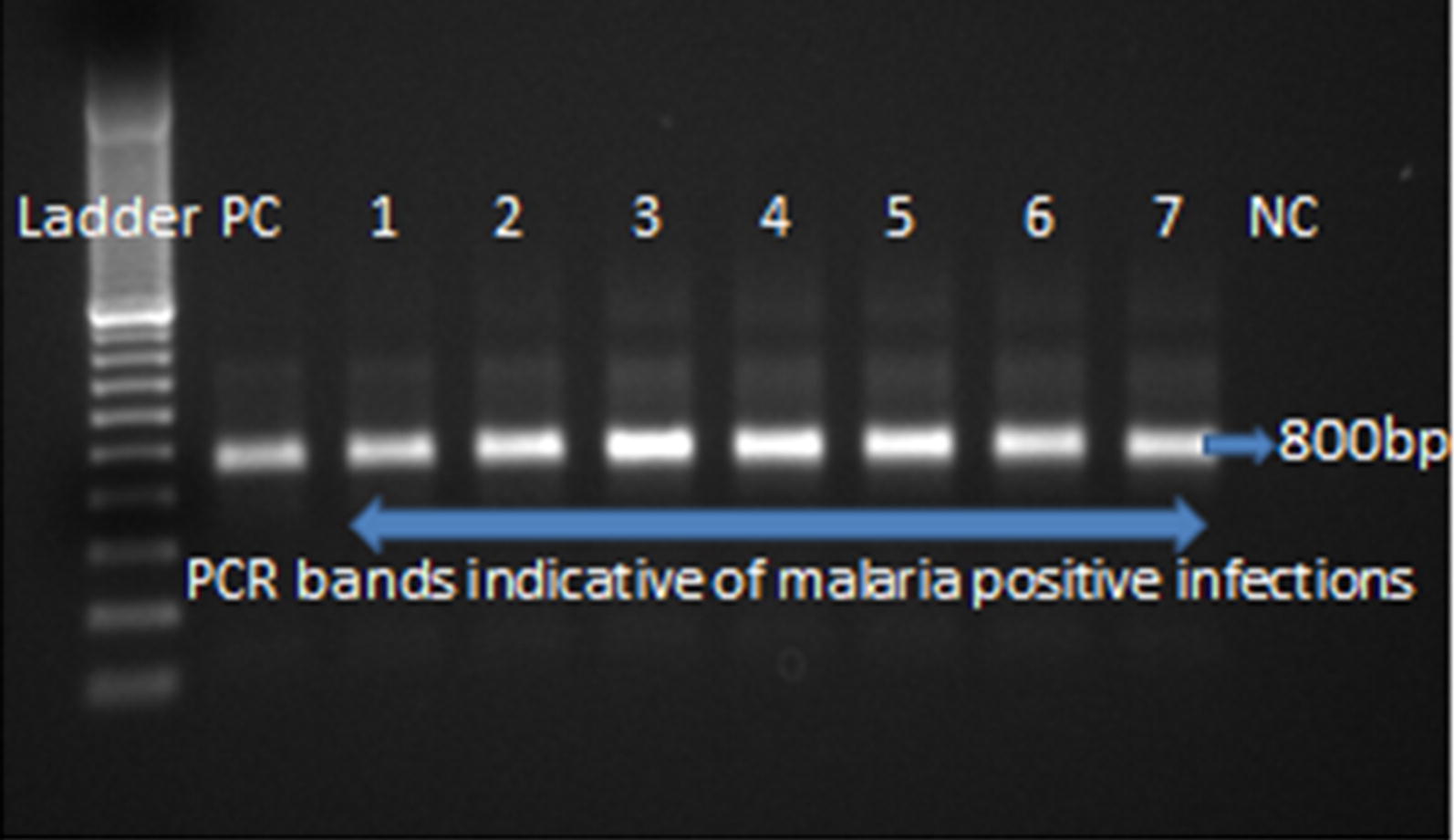
A gel showing PCR positive samples. First lane—50 bp Ladder; lane PC—Positive control; lanes 1, 2, 3, 4, 5, 6, 7—Positive samples indicating the presence of malaria infections; lane NC—negative control

There were 47 detected malaria infections with LAMP using DBS as the DNA source, two times more than those obtained with RDTs. These were detected with sensitivity, specificity, PPV and NPV of 95.50, 99.92, 99.56, and 99.92%, respectively. This is comparable to LAMP results using RDTs as the DNA source. There were 43 detected malaria infections from DBS with nPCR, 1.9 times more than those obtained with RDTs, similar to LAMP (Fig. 1).

There were 2 positive infections, picked up by LAMP and nPCR using DBS as the DNA source, that was negative by LAMP when RDTs were used as the DNA source. In addition, LAMP detected 2 malaria infections that were not detected by the gold standard nPCR with DBS as the DNA source.

## Discussion

As countries move towards elimination, interventions to halt malaria transmission will require more accurate detection of all malaria infections, both asymptomatic and symptomatic, with highly sensitive tools [5, 12, 13]. The study examined the diagnostic performance of RDT and LAMP using DNA extracts from both RDT and DBS. The study found RDT to be insensitive in detecting low density asymptomatic infections and LAMP to have similar performance to nPCR when performed on both RDT extracts and DBS.

RDTs miss community infections [14, 15]. Similarly to other studies [16], the number of infections detected by RDT nearly doubled when LAMP was used with n-PCR as the reference, as with this study [17]. This is likely due to the low detection threshold of RDT compared to LAMP with a detection limit four times higher than RDT which makes LAMP more suited for the detection of sub-patent infections [8, 16]. LAMP has a high sensitivity because, like n-PCR, it amplifies the signal (DNA) for detection compared to RDT that detects antigens in the blood. In addition to the high sensitivity of LAMP, it had a short turnaround time for results [18, 19]. The turnaround time for results for LAMP was an 8th of the turnaround time for n-PCR. LAMP has a basic benchtop preparation process that makes it an appropriate diagnostic tool, even for low resource settings in health facilities, compared to nPCR which requires working on ice and a PCR cabinet to avoid contamination. LAMP also requires minimal set-up in terms of equipment and training, and therefore it can be scaled up easily compared to nPCR.

This study examined and compared the diagnostic performance of LAMP from DNA extracts from RDTs and LAMP because samples for further molecular analysis are difficult to obtain as the number of cases decreases. Furthermore, it would be operationally convenient and cheaper if RDTs that are already routinely used by health workers could be used for further molecular research. The results show comparable results when RDT and DBS samples are used as the sources of DNA. This could provide more samples without the extra step to collect DBS, especially in low transmission setting where samples are scarce.

LAMP detected two additional positives that were missed by nPCR but it missed one infection that was detected by nPCR. These findings could mean that LAMP has false positives or it could mean that LAMP is more sensitive. In Thailand, there were reports of LAMP giving 9 false positives (n = 487) using nPCR as the gold standard [9]. Also, results were found in Ethiopia in a study carried out by Sema et al., where LAMP had one more positive than nPCR [17]. This may be because LAMP has primers that target mitochondrial DNA that has a higher copy number than the 18 s rDNA targeted by primers used in n-PCR [20–22]. In addition to this, LAMP uses a more robust polymerase, *Bst*, which is less affected by inhibitors that affect the reaction efficiency in n-PCR, which uses the *Taq* polymerase [23, 24]. In addition, LAMP is a low-cost alternative to PCR (no expensive thermocyclers required) with comparable sensitivity to n-PCR (about 70 US$0.70 per sample compared to US$5-7 per sample using DNA amplification by real-time PCR). LAMP can be run on a heat block that has several uses in the laboratory, including DNA extractions, incubation and activation of cultures, enzyme reactions and blood urea nitrogen determinations. LAMP is also used to detect other infectious diseases, such as tuberculosis and sleeping sickness, as an alternative to PCR. However, LAMP is currently more expensive than RDT, therefore, is likely to be used for quality control and detection of malaria hotspots. Beyond malaria, LAMP has been shown to be at least equally useful in diagnosis and detection of pathogens using only a heating block after DNA extraction. WHO has recommended the use of Tuberculosis LAMP (TB-LAMP\ as a replacement for microscopy for the diagnosis of pulmonary tuberculosis in adults with signs and symptoms of tuberculosis [25]. Microbiological water quality has been determined using DNA extracts from environmental waters to detect human enteric pathogens, which are a threat to public health, using only a heating block [26].

The diagnostic evaluation test gives an indication and comparison of how effective a diagnostic tool is compared to other diagnostic tools. The parameters measured by the evaluation test are sensitivity, specificity, PPV and NPV. The results from the diagnostic evaluation test show that the probability of detecting a malaria infection is quadrupled with the use of LAMP rather than RDT [27]. Therefore, the use of LAMP becomes important in RACD in order to detect sub-patent infections that are missed by RDT as a result of their poor sensitivity at low parasite density.

## Conclusion

When conducting RACD for malaria the majority of malaria infections are asymptomatic and infections are of lower density than symptomatic cases, therefore LAMP, conducted on either stored RDTs or DBS samples, performed better than RDT. Although LAMP requires minimum equipment, training and preparation making it suitable for low resource settings and operationally convenient, malaria programmes may still choose to use RDT to inform immediate clinical care of those testing positive. New, more sensitive RDTs are now in development but it is yet to be seen how they compare to LAMP and other molecular tests in the field.
